# Novel *NPC1* mutations with different segregation in two related Greek patients with Niemann-Pick type C disease: molecular study in the extended pedigree and clinical correlations

**DOI:** 10.1186/s12881-017-0409-4

**Published:** 2017-05-04

**Authors:** Evangelia Bountouvi, Anna Papadopoulou, Marie T. Vanier, Georgia Nyktari, Spyridon Kanellakis, Helen Michelakakis, Argyrios Dinopoulos

**Affiliations:** 1 Third Department of Pediatrics, Athens University Medical School, University General Hospital “Attikon”, 1 Rimini Str, 12464 –Haidari Athens, Greece; 2 Laboratoire Gillet-Mérieux, Groupe Hospitalier Est, Hospices Civils de Lyon, Lyon, France; 3 Department of Nutrition and Dietetics, Harokopio University, Kallithea, Athens, Greece; 4 Department of Enzymology and Cellular Function, Institute of Child Health, Athens, Greece

**Keywords:** Haplotype, Kindred, Miglustat, Niemann-Pick type C disease (NPC), Mutation, Polymorphisms, Therapy

## Abstract

**Background:**

Niemann-Pick type C disease (NPC) is an autosomal recessive, neurovisceral, lysosomal storage disorder with protean and progressive clinical manifestations, resulting from mutations in either of the two genes, *NPC1* (~95% of families) and *NPC2*. Contrary to other populations, published evidence regarding NPC disease in Greece is sparse.

**Methods:**

The study population consisted of two Greek NPC patients and their extended pedigree. Patients’ clinical, biochemical, molecular profiles and the possible correlations are presented. Genotyping was performed by direct sequencing. Mutations’ origin was investigated through selected exonic *NPC1* polymorphisms encountered more frequently in a group of 37 Greek patients with clinical suspicion of NPC disease and in a group of 90 healthy Greek individuals, by the use of Haplore software.

**Results:**

Two novel *NPC1* mutations, [*IVS23 + 3insT (c.3591 + 3insT*) and p. K1057R *(c.3170A > C)*] were identified and each mutation was associated with a specific haplotype. One of the patients was entered to early treatment with miglustat and has presented no overt neurological impairment after 11.5 years.

**Conclusions:**

The splicing mutation *IVS23 + 3insT* was associated in homozygocity with a severe biochemical and clinical phenotype. A possible founder effect for this mutation was demonstrated in the Greek Island, as well as a different origin for each novel mutation. Longitudinal follow-up may contribute to clarify the possible effect of early miglustat therapy on the patient compound heterozygous for the two novel mutations.

## Background

Niemann-Pick type C disease (NPC) (OMIM257220) is a rare lethal lysosomal storage disorder characterised by impaired lipid trafficking and subsequent intracellular accumulation of a wide spectrum of lipids, including unesterified cholesterol, several glycosphingolipids, sphingomyelin, sphingosine and bis (monoacylglycero) phosphate. A different pattern of stored lipids is identified in neuronal and nonneuronal tissues [1, 2]. The disease follows an autosomal recessive inheritance pattern with an estimated prevalence of 1:90.000–104.000 live births [1, 3, 4]. Clinical phenotype is highly protean with a constellation of non-specific or specific visceral, neurological and psychiatric symptoms, initiating at various ages from fetal to late adult life [5]. Visceral manifestations (neonatal cholestasis, hepatosplenomegaly) are frequent and usually mild early symptoms, with declining prevalence pertaining to increasing age of inaugural neurological signs [6, 7]. Except in a small subset of patients with a severe systemic perinatal rapidly fatal form, clinical course and prognosis correlate with the age of entering neurological symptomatology. On this basis, and although a clinical continuum has been described, categorization of patients defined by the age of onset of neurological symptoms is particularly useful [1, 8]. Intriguingly, recent data derived from massively parallel sequencing projects suggest the existence of a mild late-onset NPC phenotype with potential incidence of 1/19.000–36.000 [4].

Currently, miglustat (*N*-butyl-deoxynojirimycin), an inhibitor of glycosphingolipid synthesis, is the only approved specific therapy -as soon as any neurological sign arises- in Europe (2009) and other countries [8], but not in the United States. Data from clinical trials and observational studies mainly suggest that miglustat treatment does not prevent, but can at least decelerate the progression of some neurological manifestations, particularly in juvenile and adult onset forms that received the treatment from the very beginning of neurological impairment [6, 9, 10]. A protective effect of miglustat therapy on cerebellar and subcortical structure and function in humans has been proposed [11]. Nevertheless, evidence on long-term efficacy is lacking. Moreover, miglustat does not exert a substantial impact on cholesterol homeostasis, which is of paramount importance in the pathogenesis of NPC. Extensive research in the field of NPC therapy has been conducted during the last years, aiming at different aspects of the disease. Two products, 2-hydroxypropyl-ß-cyclodextrin (NCT01747135), which has shown significant promise in preclinical studies [12], and Arimoclomol (NCT02435030), a heat-shock protein 70/90 co-inducer in cells under stress [13], are currently on clinical trials.

Defects in either of the two distinct genes, *NPC1,* in most cases (~95%), and *NPC2* cause replicate clinical phenotypes, with a much higher frequency of early and severe pulmonary involvement in *NPC2* patients being the only remarkable difference. Although the exact function of NPC1 & NPC2 proteins remains elusive, it is supported that both interact in a convergent metabolic pathway regulating cholesterol and sphingolipid homeostasis and transport [2, 14].

The *NPC1* gene is highly polymorphic, with more than 400 disease-causing mutations reported so far (www.hgmd.cf.ac.uk) [3, 15]. Many NPC patients are compound heterozygotes, with a significant proportion of private mutations. However, some mutations are recurrent globally or among distinct populations: p. I1061T the most frequent one in the western world, p. P1007A (second most frequent) in German, p. R1186H in Czech Republic and p. G992W typically in patients of Nova-Scotia ancestry [3, 16–23]. Numerous *NPC1* genetic variations have also been identified in multiple studies, scattered over the whole gene [4]. High frequency for some of them has been recorded. Among them, the exonic variations c.387 T > C (rs12970899), c.2793C > T (rs1140458), c.644A > G (rs1805081), c.1926G > C (rs1788799), c.2572A > G (rs1805082) and c.3797G > A (rs1805084) have been recurrently observed in various populations in Europe, USA and Japan [16, 18, 24, 25]. In Greece, only three NPC patients, originating from two Aegean Sea Islands, have been reported so far. One of them was a compound heterozygote [p. F284Lfs*26 (c.852delT) and a 432 kb chromosomal microdeletion at 18q11–q12] [26] and the others were homozygous for the p. A1132P (c.3394G *>* C) mutation [27].

In the present work, we describe the clinical, biochemical and molecular profiles of two Greek NPC patients; a 12-year old female (Patient 1) with two novel *NPC1* mutations (*IVS23 + 3insT* and p. K1057R), and her cousin (Patient 2), a boy homozygous for the *IVS23 + 3insT* mutation. Patient 2 died at 3.5 years of age from the early infantile neurological form of the disease. On the basis of the family history, patient 1 was entered to early treatment, the response to which is discussed in detail. Genetic analysis of the extended kindred revealed a significant number of carriers, a different segregation for each mutation and a possible founder effect. Mutations’ origin was performed using the most frequent exonic polymorphisms of the *NPC1* gene, which occurred in a series of 37 Greek patients with clinical suspicion of NPC disease and 90 healthy Greek individuals.

## Methods

### Patients and Sampling

The study population consisted of two NPC patients and their relatives; 75 members of 3 pedigrees. The pedigrees were constructed based on oral information and questionnaires completed by the parents of each nuclear family of the extended kindred.

### NPC Patients

Our proband (Patient 1) is a 12-year-old female, the first child of phenotypically healthy non-consanguineous parents, originating from a Greek island. She was born at 39 weeks gestation age, with a birth weight of 3.2 kg (47th percentile), via caesarean section. Both pregnancy and perinatal history were without complications. At 20 days of age the neonate was admitted to the Neonatal Unit for the evaluation of icterus and hepatosplenomegaly.

Initial laboratory investigation was suggestive of neonatal hepatitis with elevated serum transaminases (SGOT 270U/L, SGPT 69U/L), γ-GT (91U/L), ALP (722U/L) and total bilirubin (193.2 umol/L), with a conjugated fraction of 77% (148.8 umol/L). Further investigation of neonatal cholestasis and comprehensive metabolic work-up were negative, except for elevated chitotriosidase levels (390nmoles/hr/ml; normal range 0–150 nmoles/ml/h). Bone marrow aspiration demonstrated storage cells compatible with NPC disease. Neurological evaluation revealed mild head lag, mild truncal hypotonia and mild upper limb hypertonia.

Niemann-Pick type C diagnosis was established at 4 months of age by biochemical and cytochemical studies on cultured skin fibroblasts. Filipin staining revealed a massive accumulation of unesterified cholesterol in perinuclear vesicles, and the early rate of Low Density Lipoprotein (LDL)-induced cholesterol esterification was almost nil (20 pmol/mg protein/4.5 h, normal values: 2950 ± 1200 pmol/mg/4.5 h) (classical biochemical subtype of the disease) [28].

At 7.5 months of age, substrate reduction therapy with miglustat was introduced, based on infant’s mild hypotonia and mainly on the worrisome family history of a relative deceased from the severe infantile form of the disease (see below). Cholestatic jaundice subsided slowly at 6 months of age but hepatosplenomegaly persisted, albeit mild, with a waxing and wane course, consistent with the usual progress of visceral symptomatology. Hypotonia completely subsided at around 20 months of age and independent walking was acquired by 16 months of age. The rest of physical and psychomotor development evolved according to her chronological age. Findings of neurophysiologic, neuroradiologic and ophthalmological studies remain within normal range. All these years neurological examination has revealed no pathological findings, apart from slight clumsiness firstly noticed at the age of 20 months. This mild developmental coordination disorder, without overt neurological signs (reflexes, tandem gait, one leg stand and cerebellar tests are within normal range), is improving overtime and therefore, it could be considered as an innate trait rather than a manifestation of the disease. Generally, treatment with dose adjusted to body surface has been well tolerated, apart from occasional soft stools that could be overcome by a lactose-free diet. Currently, at the age of 12 years, her weight is 62 kg, her height 169.5 cm, she has normal menses (menarche at 10 years of age) and leads a common life, successfully attending normal school and extracurricular activities such as, drawing and exercising (ballet, swimming and volleyball).

As mentioned above, the early clinical and biochemical diagnosis of patient 1 was expedited owing to a previously diagnosed affected cousin who had died in 1990 at the age of 3.5 years. Patient 2, a male, had been born at term to phenotypically healthy parents sharing a common origin, after an uneventful pregnancy. He had been hospitalized after birth due to cholestatic jaundice, which subsided. However, the patient progressively developed the severe early infantile neurological form of the disease with generalised hypotonia, profound developmental motor delay and rapid neurological deterioration. Diagnosis of NPC was established at 2 years of age by biochemical/cytochemical studies on cultured skin fibroblasts, showing the classical biochemical subtype of the disease [28]. The genetic defects of both patients were investigated later on.

### Haplotype analysis-*NPC1* gene variations

In lack of published evidence regarding the type and frequency of *NPC1* variations in Greek population, the study of mutations’ origin was based on the data derived from the comparison of polymorphisms between 37 Greek patients with clinical suspicion of NPC disease (SG) and 90 healthy Greek individuals (CG), which had been collected in a 4-year period (May 2011 to May 2015). The five exonic variations encountered more frequently were further studied in the 75 relatives of NPC patients 1 and 2. Haplotype analysis was performed by the use of Haplore software [29].

### Genotyping

Sequencing of *NPC1* and *NPC2* genes (exons 1–25 and 1–5, respectively, and intron-exon boundaries) was performed at the Research Laboratory of the Third Department of Pediatrics in Athens and at the Gillet-Merieux Laboratory in Lyon, France (patient 1). For studies performed in Greece, genomic DNA was isolated from peripheral blood samples using the GFX Genomic Blood DNA Purification kit (Amersham, Biosciences), according to the manufacturer’s instructions. Investigation for genetic alterations was performed by Polymerase Chain Reaction (PCR) and direct sequencing of PCR products in an ABI genetic analyzer (310 Applied Biosystems, UK), as previously described with minor modifications [30, 31]. Targeted detection of the index case/parental mutations and of the selected polymorphisms was performed in participants-members of the patients’ pedigree and a control group of 90 Greek individuals.

## Results

### NPC Patients and Pedigree

Patient 1 was found a compound heterozygote for one missense p. K1057R *(c.3170A > C)*, in exon 21, and one splicing mutation *IVS23 + 3insT* (*c.3591 + 3insT*) in exon 23 splice site. The splicing mutation leads to transcriptional splicing error and subsequent deletion of exon 23. Parental genetic testing revealed a maternal origin of the p. K1057R mutation and a paternal origin of the *IVS23 + 3insT* one. Molecular analysis on the parental *NPC1* genes of the deceased patient (Patient 2) revealed in both the same splicing mutation (*IVS23 + 3insT).* Consequently, homozygosity for an *IVS23 + 3insT* mutation was indirectly disclosed to be Patient’s 2 genetic cause of the NPC phenotype. Neither mutation was detected in 90 unaffected Greek individuals nor was reported to our knowledge.

**Fig. 1:**
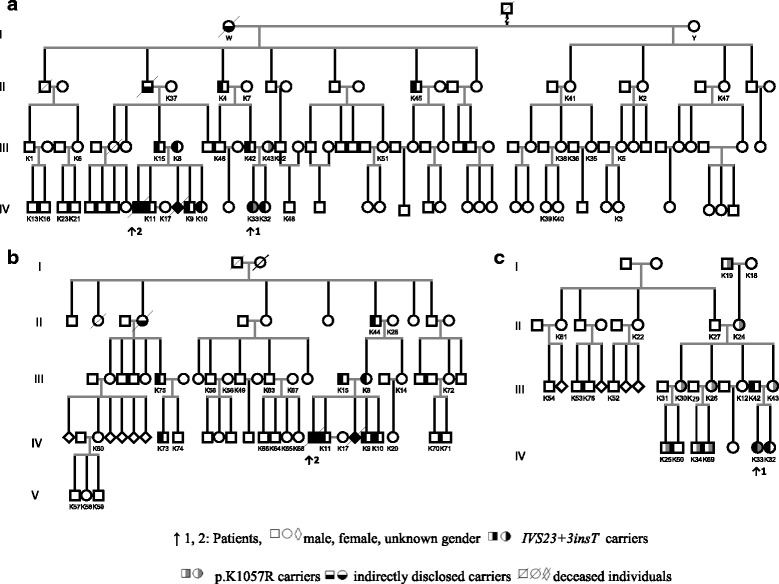
Genealogical trees **a** Fathers’ pedigree **b** Patient’s 2 maternal pedigree **c** Patient’s 1 maternal pedigree

Patient 1 and 2 are related as depicted in Fig. 1a. Maternal pedigrees of Patient 2 (K8) and Patient 1 (K43) are illustrated on Fig. 1b and c, respectively. No consanguinity was reported between these three pedigrees. Among the 75 individuals screened, 40 were male (47.4%) and 36 female (52.6%). Among the 20 mutation carriers (26.7%), 12 harbored the *IVS23 + 3insT* mutant allele and 8 owned the *K1057R* mutation.

Overall, the *IVS23 + 3insT* mutation was identified in members of the fathers’ and Patient’s 2 mother’s pedigrees (12/55, 21.8%). All individuals of these pedigrees (Fig. 1a and b) shared a common geographical origin from a region of Chania County on Crete Island. Patient 2 parents and his three siblings were all carriers of the above mutation, whereas one pregnancy was terminated due to positive prenatal testing for NPC. Ancestor Z was married twice and we assume that subject W carried the *IVS23 + 3insT* mutant allele since no descendant of subject Y was found to own the mutation. *K1057R* mutation was detected only in members of the maternal pedigree of Patient 1 (8/21, 38.1%) (Fig. 1c) and was identified to descend from an ancestor with origin from Asia Minor. This pedigree could not be investigated in depth, as no other relatives resided on the island.

### NPC1 Polymorphisms and Haplotype Analysis

Nine genetic variations classified as benign polymorphisms were identified in the exonic coding region and eleven in the intronic flanking regions of *NPC1* in the SG. Two synonymous variations c.387 T > C (rs12970899) and c.2793C > T (rs1140458) and four non-synonymous coding polymorphisms c.644A > G (rs1805081), c.1926G > C (rs1788799), c.2572A > G (rs1805082) and c.3797G > A (rs1805084) were the more frequent *NPC1* exonic polymorphisms in the SG and in the CG (unpublished data). These were further studied in Patient 1 and her pedigree. The distribution of these genotypes and allelic frequencies in the members of the NPC pedigrees is presented in Table 1.

**Table 1:** Distribution of NPC1 polymorphism genotype and allelic frequency among members of the extended pedigree (*N* =76)

Genetic variations	Genotypes detected by direct sequencing			Allelic frequency	
c.387 T > C (p. Y129Y)	TT	TC	CC	T	C
	29 (38.2)	39 (51.3)	8 (10.5)	97 (63.8)	55 (36.2)
c.644A > G (p. H215R)	AA	AG	GG	A	G
	44 (57.9)	29 (38.2)	3 (3.9)	117 (77.0)	35 (23.0)
c.1926G > C (p. M642I)	GG	GC	CC	G	C
	4 (5.3)	36 (47.4)	36 (47.4)	44 (28.9)	108 (71.1)
c.2572A > G (p. I858V)	AA	AG	GG	A	G
	2 (2.7)	65 (87.8)	7 (9.5)	69 (46.60)	79 (53.4)
c.2793C > T (p. N931N)	CC	CT	TT	C	T
	32 (35.2)	37 (48.7)	7 (9.2)	100 (65.8)	52 (34.2)
c.3797G > A (p. R1266Q)	GG	GA	AA	G	A
	69 (93.2)	5 (6.8)	0	143 (96.6)	5 (3.4)

Figures in parenthesis are percentages

Haplotype analysis in the carriers of the two novel mutations revealed that each mutation was associated with a specific haplotype; *IVS23 + 3insT* with (+,−,+,−) haplotype and *c.3170A > C* mutant allele with (−,−,−,−) haplotype for the tested SNPs; rs12970899, rs1805081, rs1788799 and rs1140458 respectively. Polymorphisms rs1805082 and rs1805084 were excluded since Hardy-Weinberg equilibrium criterion was not met for the first one and the frequency of the second one was only 3.4% in this group.

## Discussion

The genetic investigation of our NPC Greek patients revealed two novel *NPC1* mutations; the splicing mutation *IVS23 + 3insT* and the missense mutation p. K1057R. Patient 1 is a compound heterozygote for the above mutations and has displayed essentially no neurological symptomatology till the age of 12. Her cousin (Patient 2), with a homozygocity for the *IVS23 + 3insT* mutation, had suffered from the most severe neurological form of the disease and died early, at 3.5 years of age, indicating that this mutation is very deleterious. Mutations in the splice site of exon 23 affect the extracellular carboxy-terminal part of NPC1 protein, a moiety possibly susceptible to deleterious alterations, since five different splicing mutations have been reported so far (*IVS23 + 5G > A, IVS23 + 1G > A –*recurrent in Portugal-*, IVS23 + 3G > C, IVS23 + 4delA* and *IVS23 + 4insG*) [16, 18, 20, 23]. Molecular studies on total RNA extracted from cultured skin fibroblasts of NPC patients, revealed the formation of rather identical abnormal spliced transcripts of different sizes with even complete skipping of exon 23 [16, 23], as in our case. In homozygous state, the above mutations have been demonstrated to lead to functionally unstable proteins [16] and, including the novel mutation *IVS23 + 3insT* described herein, to correlate biochemically with severe impaired intracellular cholesterol trafficking (classical filipin pattern and very low LDL-induced exogenous cholesterol esterification rate) [16, 20, 23]. The clinical phenotype of three other previously reported patients with homozygous mutations at the same site [16, 23] was also very severe.

The second, missense mutation p. K1057R, consists of a point alteration at exon 21. This novel mutation resides in the cysteine-rich luminal loop L (aa 854–1098) between TM-8 and TM-9 of NPC1 protein and is predicted to perturb its function. Indeed, integrity of loop L has been demonstrated to be essential for proper cholesterol trafficking, while its zinc-binding activity indicates involvement in unloading lysosomal cargo [32]. What is more, a high number of *NPC1* mutations (1/3 of the missense mutations) is clustered in this highly conserved among most NPC1 orthologues loop [18, 33], indicating its functional significance [34]. The deleterious impact of this genetic alteration was further supported by its absence from the 90 Greek healthy subjects and also from the members of pedigrees 2a and 2b. Missense mutations in the cysteine-rich domain have been associated with both mild and severe alterations in cholesterol transport [18, 20, 35]. It has been proposed that in compound heterozygous patients one variant mutant allele is possibly sufficient for maintaining a variant biochemical phenotype [16, 18, 23, 34, 36]. The p. K1057R allele is likely associated with a classical biochemical pattern, as patient 1, carrier of *IVS23 + 3insT* and p. K1057R alleles, exhibits a classical biochemical profile.

Although striking intracellular cholesterol abnormalities are typically observed in the early infantile neurological form of the disease, whereas a “variant” biochemical phenotype is overrepresented in the adult form, clinical severity does not necessarily correspond to the degree of inhibition in intracellular LDL-cholesterol processing. Indeed, about half of the patients with an adult onset neurological form exhibit a typical filipin staining profile [36]. The missense mutation p. A1054T, adjacent to our p. K1057R mutation, has been correlated with early neurological disease and undetectable protein in immunobloting studies [35]. But since the p. K1057R mutation has not been expressed and studied by immunocytochemistry, it is currently not feasible to predict a certain genotype/phenotype correlation for this allele. A number of published patients with an adolescence/adult neurological onset is compound heterozygotes for a null and a mild *NPC1* mutant allele. On the other hand, the two patients referred in literature being compound heterozygotes for *IVS23 + 1G > A* (mutation at the same splice site) with A1035V [16] or I1061T, respectively [3], exhibited the severe infantile phenotype, despite that I1061T has been associated with a milder neurological course.

In patient 1, miglustat therapy was introduced soon after diagnosis, already at 7.5 months of age, considering mainly the natural history of the disease in her cousin and the presence of mild axial hypotonia. She has now been closely monitored for the last 11.5 years, showing good tolerance to miglustat regimen and at present, she manifests no overt neurological manifestations. The aforementioned patients with the same splicing mutation in compound heterozygosity [3, 16] had a very severe clinical course, much different from that in our Patient 1, suggesting that the early institution of substrate reduction therapy might have had an effect on our proband. However, since NPC disease presents broad heterogeneity, patient’s 1 natural history of the disease cannot be accurately determined and evidence pertaining to neurologically asymptomatic patients entering substrate therapy is elusive and sporadic [3, 37, 38], it is difficult to extrapolate any definite conclusion.

The origin of our proband from a Greek Island in association with the presence of another deceased NPC patient in her broad family prompted the investigation of the extended kindred, which revealed high prevalence of NPC carriers. This was expected since most of the pedigrees’ members are related or originate from a specific insular region. Indeed, the progenitors of the pedigrees 1a and 1b originated from two villages in close proximity and though no common ancestor could be identified, *IVS23 + 3insT* was associated with one specific haplotype. This fact strengthens the hypothesis of a founder effect, which could be further demonstrated by microsatellite analysis. On the other hand, the maternal mutation p. *K1057R* (1c pedigree) was associated with a different haplotype, which supports its different origin. Indeed, ancestors of patient 1’s mother belonged to “Diaspora” (Greeks of dispersion), who settled on the hellenic island in 1922.

A large number of *NPC1* gene variations has been identified so far [4]. The frequency and the type of the more common exonic polymorphisms observed in participants in this subgroup of Greek population are mainly in accordance with other European populations, especially Italians and Spaniards (www.ensembl.org). Of note the non-synonymous coding polymorphism rs1788799 has been associated with obesity and Alzheimer Disease/aging [39–41]. Furthermore, NPC1 protein has also been implied to be involved in the clinical entity of atheromatosis [42]. The impact of *NPC1* variations on human health has been proposed to be significant and given the limited number of functional studies in *NPC1* gene, it constitutes a promising field for further investigation [4].

## Conclusions

The novel splicing mutation *IVS23 + 3insT* reported herein is associated in homogyzous state with a severe biochemical and clinical phenotype (severe infantile form) of NPC. The new missense mutation p. K1057R is likely also associated with a classical biochemical pattern. However, it is difficult to conclude on a clinical correlation of their combination since Patient 1, carrier of the two above mutations, has been on early miglustat therapy for the last 11.5 years. The long-term follow-up of our patient may contribute to clarify the situation. Haplotype analysis suggested a possible founder effect for *IVS23 + 3insT* in the Greek Island as well as a different origin for each mutation. Note: a study on NPC in Greece has been published while our manuscript was still under evaluation [43].

## Abbreviations

CG: Control group
NPC: Niemann-Pick type C disease
PCR: Polymerase chain reaction
SG: Suspicion group
